# Anodal Transcranial Direct Current Stimulation Over the Supplementary Motor Area Improves Anticipatory Postural Adjustments in Older Adults

**DOI:** 10.3389/fnhum.2018.00317

**Published:** 2018-08-03

**Authors:** Tomonori Nomura, Hikari Kirimoto

**Affiliations:** ^1^Faculty of Rehabilitation, Niigata University of Health and Welfare, Niigata, Japan; ^2^Department of Sensorimotor Neuroscience, Graduate School of Biomedical and Health Sciences, Hiroshima University, Hiroshima, Japan

**Keywords:** anticipatory postural adjustments, center of pressure sway, motor deficit, supplementary motor area, transcranial direct current stimulation

## Abstract

We examined the influence of anodal transcranial direct current stimulation (tDCS) over the supplementary motor area (SMA) on anticipatory postural adjustments (APAs) and center of pressure (COP) sway in older adults. The study enrolled 12 healthy older adult volunteers. Subjects received anodal tDCS (2 mA) or sham stimulation over the SMA for 15 min and performed a self-paced rapid upward arm movement task on a force plate before, immediately after, and 15 min after the stimulation condition. APAs were measured as the temporal difference between activation onset in the deltoid anterior (AD) and biceps femoris (BF) muscles. The root mean square (RMS) area of COP sway, sway path length, medio-lateral mean velocity, and antero-posterior mean velocity of standing posture were also measured before and after the stimulation condition during the task. Anodal tDCS of the SMA extended APAs and decreased COP sway path length immediately after and 15 min after stimulation compared to baseline. These findings suggest that anodal tDCS over the SMA enhanced APAs function and improved postural sway during rapid upward arm movement in older adults.

## Introduction

Nitsche and Paulus (2000) were the first to report modulation of the primary motor cortex (M1) by anodal transcranial direct current stimulation (tDCS) in human subjects. Since this discovery, numerous studies have described anodal tDCS as a useful tool for improving motor performance in healthy subjects and patients with neurological disorders such as stroke hemiplegia (see review, Hashemirad et al., 2016; Kang et al., 2016). The direction of current flow determines the effects on the underlying tissue. When tDCS is applied over the primary motor cortex (M1), anodal tDCS (using the anodal electrode over M1 and the cathodal electrode over the contralateral orbit) enhances cortical excitability, which increases the amplitude of motor evoked potentials (MEPs). On the other hand, cathodal tDCS (using the cathodal electrode over M1) shows the opposite effect (Nitsche and Paulus, 2000). Yet, most previous studies applied tDCS over M1 rather than the motor association cortex. A recent study demonstrated that anodal tDCS over the supplementary motor area (SMA) promotes short-term visuomotor learning (Vollmann et al., 2013) and improves reaction times in the balance task, which is a task that requires complex planning (Hupfeld et al., 2017). In addition, anodal tDCS over SMA modulates anticipatory postural adjustments (APAs) in index finger flection tasks (Bolzoni et al., 2015) while cathodal stimulation has an inhibitory effect on APAs in rapid upward arm movements while standing (Kirimoto et al., 2013). Accordingly, anodal tDCS over the SMA may have important therapeutic utility for older adults with deteriorated balance function.

The SMA plays an important role in motor planning prior to the initiation of movement (Stephan et al., 1995; Taube et al., 2015). APAs are a representative function of the SMA. In the first report to describe APAs, it was found that activation of the postural muscles of the legs and trunk that control standing posture preceded the activation of muscles directly involved in rapid upward arm movements while standing (Belen’kii et al., 1967). APAs function is markedly reduced in patients with Parkinson’s disease; as such, the basal ganglia–subthalamic nucleus–SMA loop is thought to be involved in APAs generation (Jacobs et al., 2009). Additionally, brain function imaging studies combining functional magnetic resonance imaging (fMRI) or magnetoencephalography (MEG) and electroencephalography (EEG) have described increased excitability in the SMA, globus pallidus, putamen, and thalamus during bimanual load-lifting tasks involving APAs with healthy subjects (Ng et al., 2011).

Older adults without lesions in the basal ganglia–subthalamic nucleus–SMA loop have lower APAs than young adults (Kanekar and Aruin, 2014), and the functional degradation of APAs is associated with an increased risk of falls in older adults (Overstall et al., 1977; Hass et al., 2008). While balance is not just purely controlled via central motor processes, but also involves an interaction with cognitive function, a decrease in the connectivity of SMA – basal ganglia – thalamus, which play an important role in postural adjustment, may be considered as one cause for the reduction of APAs function. Hence, anodal tDCS over the SMA is one possible approach to restoring APA function as a fall prevention measure in aging individuals. Two previous reports have described an effect of tDCS over the SMA on APAs in healthy young adults (Kirimoto et al., 2013; Bolzoni et al., 2015), however, to our knowledge, no study has described effects on APAs function and postural regulation in older adults. If tDCS over SMA for older adults promotes the start of activation of postural muscle preceding the prime mover muscle in rapid upward arm movement tasks, it is expected to have the effect of preventing falls due to an individual’s posture change.

The aim of this study was to compare APAs and center of pressure (COP) sway at the time of a rapid upward arm movement before and after tDCS over the SMA in older adults, and to inform the potential utility of this intervention for fall prevention.

## Materials and Methods

### Subjects

We studied 12 healthy older adults [4 men and 8 women, 72.3 ± 5.3 years, mean ± standard deviation (SD)] who were able to understand and follow instructions and were without neurologic, sensory, motor, vision, or cognitive impairment. We also used a brief assessment of cognitive status, the Mini Mental Status Examination (MMSE) which patients scored on average, 29.7 ± 0.5 (mean ± SD). All subjects were strongly right-handed as determined by an Oldfield inventory score of 0.9–1.0 (Oldfield, 1971). All subjects provided written informed consent prior to the experiment. The study was conducted in accordance with the Declaration of Helsinki and the experimental protocol was approved by the ethics committee of Niigata University of Health and Welfare (approval no. 17789–170303).

### Experimental Procedures

All subjects received anodal tDCS (2 mA) or sham stimulation for 15 min in a counter-balanced order. To avoid carryover effects, each volunteer completed 2 sessions of 10 trials each on separate days that were each at least 14 days apart. Subjects performed self-paced rapid upward arm movements 10 times on a force plate before, immediately after, and 15 min after tDCS. Throughout the experiment, subjects were asked to make upward arm movements as fast as they could, and to maintain a constant COP. Prior to the start of the experiment, each subject performed the task of holding a 30 s resting standing position on the COP measurement force plate, 3 times, which was measured and the average coordinates were calculated. Subjects stood on the force plate and confirmed that their own COP position was within the average coordinate on a monitor. They were then instructed to move the right arm upward and forward to shoulder level at full speed, then hold this position for 3 s. Subjects gazed at their own COP position during the task execution and we instructed them to constantly raise their arm at a maximum effort speed in the same forward direction and to take care not to change the COP position during the task execution. In order to reduce the learning effect of the assignment, the experiment was started after sufficient training before each test session. During the task, electromyography (EMG) activity was recorded from the deltoid anterior (AD) as the prime mover muscle and the biceps femoris (BF) as a postural muscle, according to previous APAs studies (Kanekar and Aruin, 2014; Kubicki et al., 2016). Additionally, an accelerometer taped to right wrist was used to evaluate movement of the arm.

### tDCS

Transcranial direct current stimulation was delivered using a direct current stimulator (Eldith; NeuroConn GmbH, Ilmenau, Germany) through a pair of saline-soaked surface sponge electrodes (anodal, 3 cm × 3 cm; cathodal, 5 cm × 7 cm). The anodal electrode was placed to cover FC1 and FC3, which corresponds to the left SMA based on the international 10–20 extended system for electrode placement, as previously reported (Vollmann et al., 2013; Hupfeld et al., 2017). This landmark was identified by measuring and marking the skull prior to electrode placement, similar to previous studies (Cui et al., 1999; Oostenveld and Praamstra, 2001; Stock et al., 2013). The cathode electrode was placed above the right supraorbital region in order to be functionally inefficient as previously shown (Nitsche and Paulus, 2000; Nitsche et al., 2007). The current intensity of tDCS was 2 mA and the duration of stimulation was 15 min with a 30 s fade-in/fade-out time. In the sham experiment, tDCS was turned off after 30 s (Gandiga et al., 2006).

### COP Recording

Subjects stood upright on a force plate (CFP400PA102RS, Leptrino, Japan) with equal weight on each foot and their eyes open. COP visual feedback was provided on a monitor 1.5 m in front of participants with a height parallel to the line of sight. On the force plate, the distance between the feet was equal to the distance between the shoulder peaks, and the bottom outside of both fifth proximal phalanges was adjusted to the same distance. Subjects were instructed to look at and maintain the displayed COP position during the rapid upward right arm movement task. Ground reaction signals were recorded at 100 Hz and low-pass filtered (20 Hz). Data were recorded and stored on a personal computer for off-line analysis (BSMLGR, Leptrino, Japan). The average COP RMS area, sway path length, medio-lateral (ML) mean velocity, and antero-posterior (AP) mean velocity were calculated for analysis.

### EMG and Acceleration Recording

Surface EMG was recorded from the right AD and BF muscles using double differential active electrodes (FSE-DEMG1, 4Assist, Japan). The skin was cleaned with alcohol and the recording and reference electrodes were placed over the center of each muscle. A ground electrode was attached to the anterior aspect of the leg over the left tibia. EMG signals were amplified (×500) and band-pass filtered (5–1,000 Hz) with an EMG amplifier system (FA-DL-140, 4assist, Japan) and digitized at 10 KHz (PowerLab, AD Instruments, Bella Vista, NSW, Australia). Data were also recorded form 3-axis acceleration sensors (FA-DL-110, 4Assist, Japan) attached to the subject’s right wrist and stored on a personal computer for off-line analysis (Chart 7, AD Instruments, Bella Vista, NSW, Australia).

### Statistical Analysis

The EMG signal baseline for each muscle was sampled over a period of 100 ms while the participant stood quietly prior to beginning any movement. The activity onset in each muscle was defined as the point at which the EMG signal reached at least 3 SD above the mean baseline for a period of at least 20 ms (Nana-Ibrahim et al., 2008). APAs were measured by computing their timing (APAt) as previously reported (Fujiwara et al., 2003). APAt was defined as the temporal difference between activation onset in the AD and BF muscles (ΔEMG onset). The coefficient of variations (CV) was calculated to confirm the stability of the change of ΔEMG onset. The calculated average value ($\bar{X}$) and SD (σ) of ΔEMG onset for each session (*CV* = σ / $\bar{X}$) was then determined. A decreasing CV with a ΔEMG onset indicated a stable ΔEMG onset. Acceleration onset was measured using the same calculation method as EMG onset based on the 1,000 ms before and after movement onset; these values were used to compute the interval of acceleration and COP. The average acceleration and maximum acceleration on the y axis were measured, and the accuracy of the upward arm movement was evaluated. ΔEMG onset, average acceleration, maximum acceleration, RMS area, sway path length, ML mean velocity, and AP mean velocity were calculated the average data of the 10 trials each stimulation condition and normalized by the average values obtained before the stimulation condition. Parameter values taken before tDCS, immediately after tDCS, and 15 min after tDCS were compared with a two-way repeated-measures analysis of variance (ANOVA) (intervention [sham, anodal]) × (time [before tDCS, immediately after tDCS, 15 min after tDCS]). Significant differences were further analyzed with Bonferroni *post hoc* tests. All analyses were performed with IBM SPSS Statistics software version 20 (SPSS; IBM, Armonk, NY, United States) and the significance level was set at 5%.

## Results

### EMG Activity After tDCS Over the SMA

**FIGURE 1:**
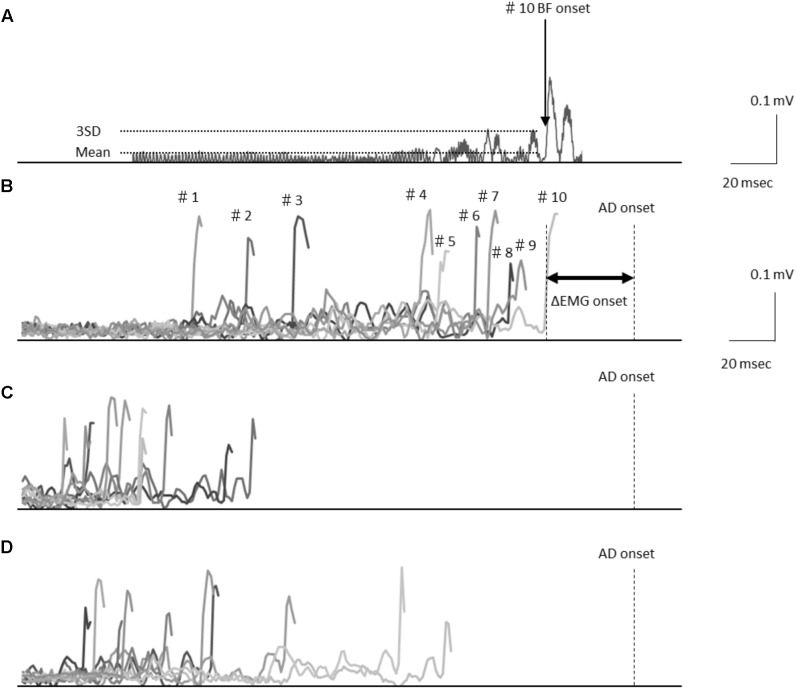
**(A)** Electromyography waveforms in the biceps femoris (BF) during a self-paced rapid shoulder flexion task for a representative case. BF onset was defined as the point at which the EMG signal reached at least 3 standard deviations above the mean baseline. Data are shown representing the baseline stimulation condition **(B)**, immediately after stimulation **(C)**, and 15 min after stimulation **(D)** recorded from the representative subject. Electromyography waveforms were presented for the baseline stimulation condition in 10 trials of BF onset. Latency differences (ΔEMG onset) were calculated by subtracting the time of EMG burst onset of the BF (BF onset) from that of the activation onset of the deltoid anterior muscle (AD) (AD onset).

**FIGURE 2:**
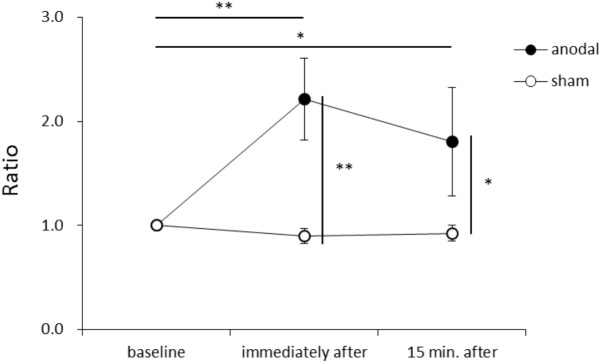
Serial changes in ΔEMG onset before, immediately after, and 15 min after anodal or sham transcranial direct current stimulation (tDCS) over the supplementary motor area (SMA). ΔEMG onset was calculated by subtracting the time of EMG burst onset of the biceps femoris from that of the activation onset of the deltoid anterior muscle. Data were normalized to average value at baseline (mean ± standard error of the mean). ^∗^*p* < 0.05, ^∗∗^*p* < 0.01.

**FIGURE 3:**
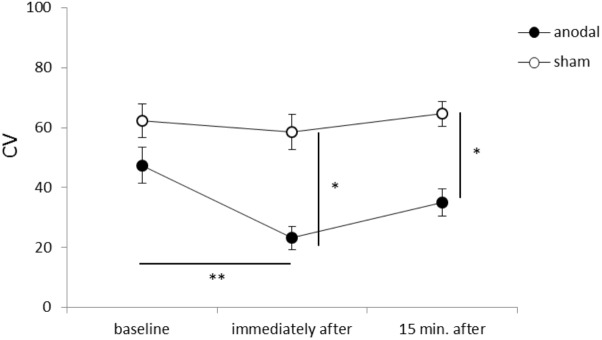
Serial changes in the percentage coefficient of variation (CV) before, immediately after, and 15 min after anodal or sham tDCS over the SMA (mean ± standard error of the mean). ^∗^*p* < 0.05, ^∗∗^*p* < 0.01.

**Figure 1** shows representative EMG waveforms in the BF during the self-paced rapid shoulder flexion task before, immediately after, and 15 min after anodal tDCS over the SMA. ΔEMG onset was extended after anodal tDCS compared to baseline. A two-way repeated measures ANOVA of ΔEMG onset revealed significant main effects of intervention [*F*_(1,11)_ = 10.267, *p* = 0.008, η^2^= 0.483, 1–β = 0.83], time [*F*_(2,22)_ = 6.595, *p* = 0.006, η^2^= 0.375, 1–β = 0.74], and the intervention × time interaction term [*F*_(2,22)_ = 11.293, *p* = 0.002, η^2^= 0.432, 1–β = 0.81] (**Figure 2**). A *post hoc* analysis revealed significant differences between anodal and sham tDCS both immediately after (*p* = 0.005) and 15 min after stimulation (*p* = 0.020). There were also significant differences between baseline and immediately after stimulation (*p* = 0.006) and between baseline and 15 min after stimulation for anodal tDCS (*p* = 0.025). **Figure 3** shows percentages of the coefficients of variation (CVs) in ΔEMG onset. An ANOVA of *CV*-values revealed significant main effects of intervention [*F*_(1,11)_ = 6.187, *p* = 0.032, η^2^= 0.382, 1–β = 0.62] and time [*F*_(2,22)_ = 3.982, *p* = 0.035, η^2^= 0.285, 1–β = 0.65]. A *post hoc* analysis showed significant differences between anodal and sham tDCS both immediately after (*p* = 0.022) and 15 min after stimulation (*p* = 0.017). There was also a significant difference between baseline and immediately after stimulation for anodal tDCS (*p* = 0.002).

### Upward Arm Movement Acceleration

**FIGURE 4:**
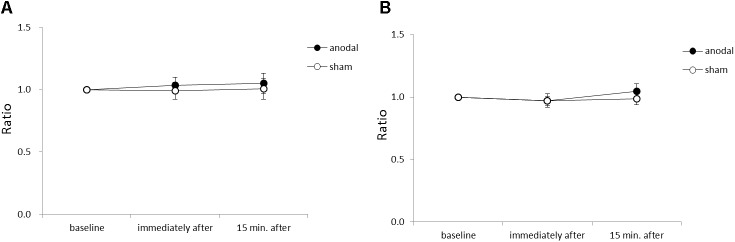
Serial changes in average acceleration and maximum acceleration in the *y*-axis before, immediately after, and 15 min after anodal or sham tDCS over the SMA. **(A)** Normalized values of average acceleration in the *y*-axis. **(B)** Normalized values of maximum acceleration in the *y*-axis. All data reflect the mean ± standard error of the mean.

**Figure 4** shows the average acceleration (A) and maximum acceleration (B) of rapid upward arm movements in the y-axis for each time-point in each stimulation condition. There were no significant between-group or within-group differences.

### COP Sway After tDCS Over the SMA

**FIGURE 5:**
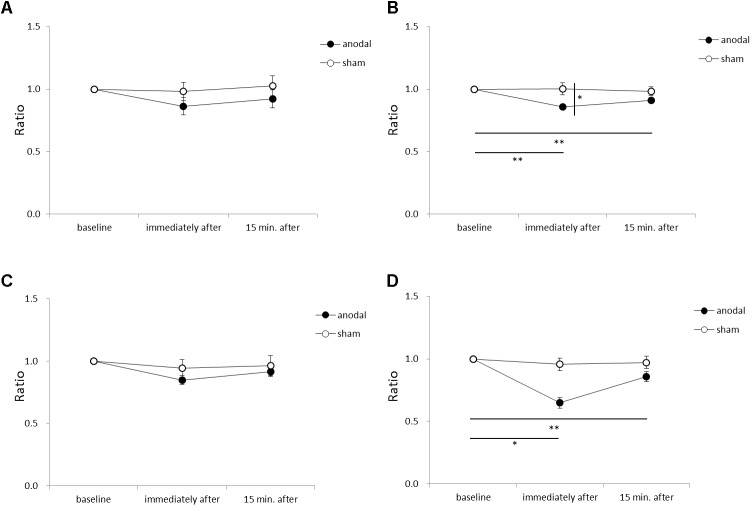
Serial changes in root mean square (RMS) area, sway path length, medio-lateral (ML) mean velocity, and antero-posterior (AP) mean velocity before, immediately after, and 15 min after anodal or sham tDCS over the SMA. **(A)** Normalized RMS area values. **(B)** Normalized sway path length values. **(C)** Normalized ML mean velocity values. **(D)** Normalized AP mean velocity values. All data reflect the mean ± standard error of the mean. ^∗^*p* < 0.05, ^∗∗^*p* < 0.01.

**Figure 5** shows the RMS area, sway path length, ML mean velocity, and AP mean velocity for each time-point in each stimulation condition. An ANOVA of sway path length revealed significant main effects of intervention [*F*_(1,11)_ = 6.449, *p* = 0.028, η^2^= 0.370, 1–β = 0.74], time [*F*_(2,22)_ = 7.085, *p* = 0.004, η^2^= 0.392, 1–β = 0.82], and the intervention × time interaction term [*F*_(2,22)_ = 4.197, *p* = 0.029, η^2^= 0.276, 1–β = 0.60]. A *post hoc* analysis showed significant differences between anodal and sham tDCS immediately after stimulation (*p* = 0.022), between baseline and immediately after stimulation for anodal tDCS (*p* < 0.001), and between baseline and 15 min after stimulation for anodal tDCS (*p* < 0.001). An ANOVA of AP mean velocity revealed a significant main effect of time [*F*_(2,22)_ = 5.713, *p* = 0.010, η^2^= 0.342, 1–β = 0.60]. A *post hoc* analysis showed significant differences between baseline and immediately after stimulation for anodal tDCS (*p* = 0.004), and between baseline and 15 min after stimulation for anodal tDCS (*p* = 0.012). There were no significant between-group or within-group differences in RMS area or ML mean velocity.

## Discussion

The present study demonstrated that anodal tDCS over the SMA extended ΔEMG onset time for the AD and BF muscles during a rapid upward arm movement task in healthy older adults. Additionally, the CV of ΔEMG onset was decreased after anodal tDCS compared to sham stimulation. Further, COP sway path length was decreased immediately after and 15 min after tDCS compared to baseline. These findings suggest that tDCS over the SMA enhanced the timing of postural regulator muscle activity preceding rapid upward arm movements and strengthened stable execution of the APAs function in healthy older individuals.

Previous studies have reported that APAs are changed by the COP position before the start of motion (Fujiwara et al., 2003) and by acceleration in the upward arm movement task (Lee et al., 1987). When upward arm movements are made slowly with backward positioning of the body’s center of gravity, forward movement of the body center of gravity is estimated to be small, and thus APAs time is shortened. In this study, subjects were asked to execute upward arm movements as fast as they could while maintaining a constant COP. Importantly, average acceleration of the rapid upward arm movement did not vary between before and after tDCS. Therefore, changes in ΔEMG onset and its CV as well as COP sway path length can be attributed to anodal tDCS over the SMA rather than confounding factors such as changes in COP position and decreased acceleration of upward arm movements over time.

As described in other postural control tasks, older adults exhibit delays in the onset of anticipatory postural EMG activity compared to young adults (Kanekar and Aruin, 2014). In our previous study, the APAs time of young adults was 60 ms in a self-paced rapid upward arm movement task (Kirimoto et al., 2013). In this study of older subjects, the APAs time was extended from 36 ms pre-stimulation to 81 ms immediately after stimulation. This result shows that APAs time of older subjects pre-stimulation is delayed more than in the young subjects and approaches APA time of young subjects immediately after stimulation. Studies of young healthy adults suggest that anodal tDCS over the SMA is associated with improvements in motor skill (Vollmann et al., 2013). Further, SMA activation appears to be highly correlated with motor skill learning, suggesting an important role of the SMA in the acquisition of new motor skills (Lefebvre et al., 2012). Our results are consistent with these studies and suggest that anodal tDCS improved the stability of the postural control during rapid upward arm movement. The stability of posture control is meaningful for older adults, as many activities of daily living are conventionally performed in a stable standing position.

Our results also showed positive effects of anodal tDCS on the COP sway path length, corresponding to improved motor performance in postural control. The COP sway path length is increased in older adults compared to young adults (Benjuya et al., 2004). Additionally, fallers show a significantly higher COP sway path length compared to non-fallers (Melzer et al., 2004), and the COP sway path length is an independent factor predicting falls in older adults (Johansson et al., 2017). Taken together, improvements in the COP sway path length by anodal tDCS over the SMA may decrease the likelihood of falling in older adults.

Transcranial direct current stimulation is a non-invasive technique that allows the modulation of cortical excitability in humans (Nitsche and Paulus, 2000; Priori, 2003). It is thought that neuronal cell membranes below the anode are depolarized while those below the cathode are hyperpolarized, leading to increases and decreases in cortical excitability, respectively (Nitsche and Paulus, 2000; Antal et al., 2017; Lefaucheur et al., 2017). The cathode electrode is most effective when placed on the forehead on the contralateral side (Nitsche and Paulus, 2000). However, in any place it may affect the cortex beneath the cathode electrode (Schambra et al., 2011). It may also be necessary to consider placing the reference electrode outside the head. Numerous studies have reported the improvement of various motor functions by tDCS over M1 in healthy subjects and patients on the premise of this hypothesis (Hashemirad et al., 2016; Kang et al., 2016). In this study, we selected the SMA stimuli position by using tDCS on the scalp in conformity with a previous study of SMA tDCS in young adults by Hupfeld et al. (2017). Anode tDCS can lead to improved connectivity of the SMA pathway, connectivity between SMA and M1, SMA- cerebellar connectivity (Hamada et al., 2009; Polanía et al., 2012; Hupfeld et al., 2017). We hypothesize that tDCS promoted connectivity of the SMA modulated within the APAs processing network, consistent with the implications of previous research. It is possible that reinforcement of mutual connectivity of the SMA may have a positive effect on posture adjustment improvement. In our previous study, we also reported stimulatory effects of simultaneous tDCS over the SMA and dorsal premotor cortex on distant sites including M1 and the somatosensory cortex (Kirimoto et al., 2011). Accordingly, it is possible that the modulation of areas other than the SMA responsible for generating and outputting APAs (e.g., M1) was in part responsible for the observed changes in EMG and COP parameters in older adults. Further studies are needed to elucidate the neurophysiological effects of tDCS. In particular, it is necessary to verify whether there is an effect of tDCS over the SMA on posture control and risk of falling in older adults using electrophysiological methods.

### Limitations

The sample size was small in this study which was one of the major limitations. However, in our study, we were able to examine whether the anodal tDCS can contribute to posture control for APAs functional changes due to aging of healthy older adults. These results have the potential to inform the development of anodal tDCS enhanced protocols in training. The effects of promotion of APAs function may be expected using the anodal tDCS as a condition stimulus before training. We will continue to consider protocols for older adults with balance disabilities. The findings in this study may assist with the development of enhanced protocols involving the combination of anode tDCS with exercise training and other rehabilitation protocols.

## Conclusion

The present study demonstrates that anodal tDCS over the SMA extended ΔEMG onset time, decreased the CV of ΔEMG onset time, and reduced the COP sway path length during a rapid upward arm movement task. We suggest that anodal tDCS over the SMA is an effective method for improving APAs function in older adults.

## Author Contributions

HK conceived the study and designed the experiment. TN conducted the experiments. TN and HK performed the statistical analysis and interpreted the data, wrote and approved the manuscript.

## Conflict of Interest Statement

The authors declare that the research was conducted in the absence of any commercial or financial relationships that could be construed as a potential conflict of interest.
